# Expert Consensus on Ivabradine-based Therapy for Heart Rate Management in Chronic Coronary Syndrome and Heart Failure with Reduced Ejection Fraction in India

**DOI:** 10.2174/1573403X19666230320105623

**Published:** 2023-07-17

**Authors:** J C Mohan, I Sathyamurthy, Monotosh Panja, Rajeev Agarwala, C K Ponde, A Sreenivas Kumar, Bijay Kumar Mahala, Vivek Kolapkar, R V Lokesh Kumar, Kamlesh Patel

**Affiliations:** 1 Head of Department & Senior Consultant Cardiology, Jaipur Golden Hospital, Delhi, India;; 2 Senior Interventional Cardiologist, Apollo Hospitals, Chennai, India;; 3 Senior Interventional Cardiologist, AMRI Hospitals, Kolkata, India;; 4 Head of Department and Consultant Cardiologist, Jaswant Rai Speciality Hospital, Meerut, India;; 5 Head of Department and Consultant Cardiologist, P. D. Hinduja National Hospital & Medical Research Centre, Mumbai, India;; 6 Director Cardiology & Clinical Research, Apollo Health City, Hyderabad, India; Apollo Hospitals, Hyderabad, India;; 7 Senior Consultant Cardiology, Narayana Institute of Cardiac Sciences, Bangalore, India;; 8 Medical Affairs, Lupin Limited, Mumbai, India

**Keywords:** Expert opinion, consensus, Ivabradine, β-blocker, chronic coronary syndromes, heart failure with a reduced ejection fraction

## Abstract

Heart rate is an important indicator of health and disease and the modulation of heart rate can help to improve cardiovascular outcomes. Besides β-blockers, Ivabradine is a well-established heart rate modulating drug that reduces heart rate without any hemodynamic effects. This consensus document was developed with the help of expert opinions from cardiologists across India on effective heart rate management in routine clinical practice and choosing an appropriate Ivabradine-based therapy considering the available scientific data and guideline recommendations. Based on the discussion during the meetings, increased heart rate was recognized as a significant predictor of adverse cardiovascular outcomes among patients with chronic coronary syndromes and heart failure with reduced ejection fraction making heart rate modulation important in these subsets. Ivabradine is indicated in the management of chronic coronary syndromes and heart failure with reduced ejection fraction for patients in whom heart rate targets cannot be achieved despite guideline-directed β-blocker dosing or having contraindication/intolerance to β-blockers. A prolonged release once-daily dosage of Ivabradine can be considered in patients already stabilized on Ivabradine twice-daily. Ivabradine/β-blocker fixed-dose combination can also be considered to reduce pill burden. Two consensus algorithms have been developed for further guidance on the appropriate usage of Ivabradine-based therapies. Ivabradine and β-blockers can provide more pronounced clinical improvement in most chronic coronary syndromes and heart failure with reduced ejection fraction patients with a fixed-dose combination providing an opportunity to improve adherence.

## 1 INTRODUCTION

Cardiovascular disease (CVD) is one of the prevalent causes of the increasing non-communicable disease burden worldwide. A recent Global Burden of Disease (GBD) update reported a steep rise in the prevalence from 1990 (271 million) to 2019 (523 million) and a gradual rise in the number of CVD deaths currently accounting for a total of 18.6 million in 2019 [1]. Notably, India’s contribution to CVD deaths (272 per 100000 population) is substantially higher than the global average of 235 per 100000 population [1]. It is well-established that the coronary artery disease (CAD) burden in Indian population is rising at a quick pace resulting in 4 times higher hospitalization rates due to complications of CAD compared to other ethnicities and 5-10-fold higher admission rates in young individuals (<40 years) [2]. As per the 2019 European Society of Cardiology (ESC) Guidelines, new and more precise terminology has been proposed for stable CAD as chronic coronary syndromes (CCS) [3]. The rising prevalence of heart failure (HF) is another challenge, with the world being home to an estimated 64.3 million individuals affected by HF [4]. The HF has recently been classified into three major types depending on the left ventricular ejection fraction (LVEF): HF with reduced ejection fraction of ≤40% (HFrEF), HF with a mildly reduced ejection fraction of 41-49% (HFmrEF) and HF with a preserved ejection fraction of ≥50% (HFpEF). The incidence of HFrEF as per the Trivandrum Heart Failure Registry (THFR) was around 62% of the total HF population, indicating that HFrEF is the predominant type of HF observed in the Indian population which was also associated with the highest mortality [5]. The probable reason for it is the early and high incidence of CAD which was also the aetiological factor in almost 75% of patients in THFR. Also, the mean heart rate (HR) observed in the patients with HFrEF was 99 beats per minute (bpm) despite 65% of them being on β-blockers. HR is the core indicator/marker of life and in a normal adult, HR ranges between 60 and 100 bpm. HR is the most important factor affecting myocardial oxygen demand and an increase in heart rate increases the myocardial oxygen requirement. Generally, abnormalities in HR are considered one of the independent risk factors associated with the incidence of CVD and CVD-related morbidities, mortality and adverse clinical outcomes [6-9]. Therefore, a crucial and fundamental aspect of the management of CVDs is to target the modulation of HR through appropriate therapies available. Achieving the target HR is one of the important goals, especially in the management of patients with CCS and HFrEF. With regards to HR management in CCS and HFrEF, there is a lack of Indian consensus on what should be the appropriate strategy in routine clinical settings. Considering this background, a consensus document was formulated to help physicians understand the significance of high HR in CCS and HFrEF and how HR modulating agents like Ivabradine can be helpful. The document will also aid in choosing appropriate Ivabradine-based therapy for effective HR management in routine clinical practice. To develop the consensus document, a core committee of 8 cardiologists was formulated and the methodology was finalized in a national meeting. Each of these 8 cardiologists conducted zonal meetings to discuss and seek an opinion from other expert cardiologists from different parts of India. Compilation of discussion points and opinions based on the national and zonal meetings was done to prepare a consensus document. The points for discussion included the significance of HR modulation in CVD, the role of Ivabradine as HR modulating agent, HR targets in CCS and HFrEF, and the role of Ivabradine/β-blocker combination in CCS and HFrEF. This consensus report was reviewed and finalized by the core committee members.

## 2 SIGNIFICANCE OF HEART RATE MODULATION IN CVD

Based on consistent evidence from epidemiological studies, elevated HR or resting HR (RHR) is considered a vital indicator of disease and mortality both in the general population and in patients with CCS and HFrEF [6]. Emerging evidence suggests that Indians have a higher HR with an average RHR of around 81 bpm as reported by the India Heart study and BEAT survey [10, 11]. The possible factors associated with high RHR include high prevalence (62.42%) of sympathetic overactivity (SO) and smaller heart chamber size as compared to western cohorts wherein high HR is required to maintain the cardiac output [12, 13]. This imbalance between sympathetic and parasympathetic activity has been implicated in the observed associations between raised RHR and adverse cardiac outcomes [14, 15]. Likewise, observations from previous epidemiological studies also indicated that elevated RHR was associated with increased risk of hospitalization, cardiovascular (CV) and all-cause mortality among patients with CVD [15, 16]. The BEAUTIFUL (morBidity-mortality EvAlUaTion of the If inhibitor Ivabradine in patients with coronary disease and left-ventricULar dysfunction) trial demonstrated that patients with HR ≥70 bpm as compared to those with HR <70 bpm were at a significantly higher risk for CV death (34%), hospitalizations for HF (53%), hospitalization for myocardial infarction (MI) (46%) and coronary revascularization (38%) [16]. Moreover, every 5 bpm increase in HR increased the risk of CV death by 8%, hospitalization for HF by 16%, hospitalization for MI by 7% and coronary revascularization by 8% [16], highlighting the consequences of inadequate HR control in patients with CCS. A retrospective real-world data involving patients with HFrEF showed that at the time of diagnosis and follow-up an elevated RHR (>70 bpm) was strongly associated with an increased risk of adverse outcomes, including a 36% increase in mortality, 25% increase in annual all-cause hospitalization time and 51% increase in annual hospitalization time due to HF [17]. With respect to discharge HR, Habal *et al.* demonstrated that elevated HR (>90 bpm) at discharge in patients with HF was associated with increased risk of all-cause and CV mortality at one-year with 26% increased incidence of one-month re-hospitalization due to worsening HF and 29% higher risk of CVD within one-month post-discharge [18]. Overall the evidence suggests that elevated HR acts as a risk marker as well as a risk factor across the CV disease continuum and reduction of HR to the optimal levels can help to improve prognostic outcomes in patients with CCS and HFrEF [19].

**Key Consensus Recommendations 1**

***Significance of high RHR as a risk marker and a risk factor***

- High RHR is a simple yet important predictor of adverse CV events across the CV continuum.
- In general, high RHR can be considered a risk marker in the healthy population and CCS patients whereas, in patients with CCS and systolic dysfunction, HFrEF patients, high RHR is a risk factor.
- In patients with CCS and HFrEF, controlling RHR is of utmost importance, which can be achieved with the help of β-blockers and Ivabradine.
- In individuals having high RHR without any evidence of cardiac disorders or risk factors, lifestyle modification rather than drugs can be considered.

## 3 ROLE OF IVABRADINE AS AN HR MODULATING AGENT IN CCS AND HFREF

Ivabradine is a selective HR-reducing agent that inhibits the HR regulating channel responsible for the cardiac pacemaker current, I(f). Ivabradine has a negative chronotropic effect on the sino-atrial node and it is a pure HR-lowering agent with distinct benefits in HFrEF. This unique feature also contributes to the overall favorable safety profile of Ivabradine with the absence of significant side effects associated with other HR-reducing therapies. Among patients with CCS, Ivabradine reduces oxygen demand in myocytes, reduces energy requirements, and reduces ischemia and angina; whereas in HFrEF, it improves coronary perfusion, reduces the load on the heart muscle, improves heart muscle functioning and prevents worsening of HF [20]. Ivabradine is available in India as twice-daily immediate-release and once-daily sustained-release formulations as monotherapy and also in a fixed-dose combination (FDC) with metoprolol and carvedilol. Ivabradine is indicated for the management of chronic stable angina pectoris with normal sinus rhythm and heart rate >60 bpm and in chronic HF patients (NYHA class II to IV) with systolic dysfunction and heart rate >75 bpm as an add-on to β-blockers. Additionally, it can also be a useful agent for HR reduction in patients with contraindication/intolerance to β-blockers. The recommended starting dose of Ivabradine is 5 mg twice daily [21]. In the PROFICIENT (PROlonged Release Formulation of Ivabradine OnCe-DaIly in HEart Rate ManagemeNT) study, HR was effectively maintained with Ivabradine OD dosage among those who shifted from Ivabradine twice-daily dose [22]. Once-daily dose thus can effectively maintain the HR, which may aid in improving treatment compliance. The key contraindications associated with the use of Ivabradine include hypersensitivity to Ivabradine, cardiac arrhythmias, acute myocardial infarction, severe hypotension (<90/50 mmHg), pretreatment resting heart rate <60 bpm [21], sick sinus syndrome, sino-atrial block, acute HF, pacemaker dependence, unstable angina, atrioventricular block (second/third degree), atrial fibrillation or other cardiac arrhythmias, severe hepatic insufficiency, stroke, pregnancy, lactating women, concomitant treatment with strong CYP3A4 inhibitors, CV and non-CV QT prolonging agents, potassium-depleting diuretics and non-dihydropyridine calcium channel blockers like verapamil or diltiazem. The most common adverse events associated with Ivabradine are luminous phenomena (phosphenes) and bradycardia. They are dose-dependent and related to the pharmacological effect of the drug [23]. No dose adjustments are necessary for patients with mild hepatic impairment, however, ivabradine should be cautiously used in patients with moderate hepatic impairment. Ivabradine is contraindicated in patients with severe hepatic impairment (Child-Pugh C). No dosage adjustment of ivabradine is needed for patients who have mild, moderate, or severe renal insufficiency or creatinine clearance >15mL/min [21, 23].

### 3.1 Spectrum of Clinical Evidence with Ivabradine in CCS

Numerous studies have demonstrated the efficacy and safety of Ivabradine in CCS as a monotherapy and combination therapy with other antianginal agents, including β-blockers [23]. Ivabradine has anti-anginal and anti-ischemic effects regardless of age, sex, severity of angina, revascularization status or comorbidities [23]. The BEAUTIFUL study demonstrated a comprehensive benefit of Ivabradine use in patients with stable CAD and LV dysfunction with reduced rates of hospitalization, the need for coronary revascularization and hospitalization for CV events in patients among baseline HR of ≥70 bpm [16]. In addition, a subgroup analysis involving patients who had angina at baseline, Ivabradine administration significantly reduced CV death, MI and hospitalization for HF by 24% and reduced hospitalization for MI by 42% as compared to placebo [24]. On the contrary, SIGNIFY (Study assessInG the morbidity-mortality beNefits of the If inhibitor Ivabradine in patients with coronarY artery disease) trial did not report any benefit of Ivabradine in reducing the risk of CV death or nonfatal MI among patients with stable CAD, LVEF >40% and HR ≥70 bpm [25]. Possible reasons for the lack of benefit seen in SIGNIFY trial could be i) higher mean age (65.0 years) of the study population; ii) higher initiation and maintenance dose regimen of Ivabradine (10 mg twice-daily) in 47% of patients aged <75 years may have increased risk of bradycardia events; iii) concurrent use of verapamil or diltiazem with Ivabradine was associated with increased nonfatal MI and ~3-fold increases in plasma Ivabradine levels [25, 26].

### 3.2 Guideline Recommendations in CCS

As per the 2019 ESC guidelines on CCS, Ivabradine should be considered as a second-line treatment to reduce angina frequency and improve exercise tolerance in subjects who cannot tolerate or have contraindications to or whose symptoms are not adequately controlled by β-blockers, calcium channel blockers (CCB) and long-acting nitrates. In selected patients, the combination of a β-blocker or a CCB with Ivabradine may be considered for first-line treatment based on HR [3]. The ESC 2021 HF guidelines recommended Ivabradine in HFrEF patients with persistence of CCS symptoms and HR ≥70 bpm in sinus rhythm despite using guideline-recommended/maximally tolerated doses of β-blockers [27].

### 3.3 Spectrum of Clinical Evidence with Ivabradine in HFrEF

Ivabradine has also been extensively studied in the setting of HFrEF, with SHIFT (Systolic Heart Failure Treatment with the If Inhibitor Ivabradine Trial) being the major randomized, double-blind, multicentre placebo-controlled trial involving 6558 patients with HFrEF on background β-blocker therapy and HR >70 bpm [28]. The study reported a significant benefit of Ivabradine use in terms of HR reduction and subsequent reduction of the primary composite endpoint of CV death or hospitalization for worsening HF by 18%. Patients with the highest pre-intervention HR (HR ≥77 bpm) had the greatest reduction in HR with Ivabradine and also the largest reduction in the primary composite endpoint of CV death or hospital admission for worsening HF [28]. An evaluation of the impact of background β-blocker dose on response to Ivabradine among patients with systolic HF, sinus rhythm and HR ≥70 bpm revealed that HR reduction with Ivabradine is independent of the dose of β-blocker but dependent on RHR [29]. A study by Borer *et al.* demonstrated that the addition of Ivabradine to a background guideline-based HF therapy reduced the risk of re-hospitalizations among patients with moderate-to-severe HF and LV systolic dysfunction [30]. The need for second and third hospitalization was also significantly reduced for HF during a median follow-up of 22.9 months. In addition, a post-hoc analysis of SHIFT indicated a 30% reduction in the risk of re-hospitalization in the vulnerable phase (during one-month post-discharge following HF hospitalization) [30]. These studies confirmed the benefit of Ivabradine therapy in reducing the risk of re-hospitalization for HF. Ivabradine is devoid of a negative inotropic effect and also does not share the blood-pressure-lowering effects of β-blockers, renin-angiotensin-aldosterone system (RAAS) blockers. Thus, treatment with Ivabradine might be relatively manageable due to fewer chances of adverse effects in the days or weeks following hospitalization [31].

### 3.4 Guideline Recommendations in HFrEF

The ESC 2021 and 2022 American HF guidelines have recommended Ivabradine for patients with symptomatic stable chronic HFrEF in sinus rhythm whose HR is ≥70 bpm and who are on maximum tolerated dose of β-blockers and other guideline-directed medical therapies (GDMT) [27, 32]. Similarly, the American College of Cardiology (ACC) 2021 consensus decision pathway for HFrEF recommends a 2.5-5 mg twice-daily dosage of Ivabradine in patients with HFrEF (EF ≤35%) having sinus rhythm with RHR ≥70 bpm despite maximally tolerated β-blocker dose with NYHA class II or III symptoms [33]. Moreover, the Heart Failure Association of ESC in the 2021 consensus document has provided HFrEF patient profiles suitable for Ivabradine use which include patients with low blood pressure (BP) and high HR >70 bpm; patients with low BP and HR 60-70 bpm; patients with normal BP and high HR >70 bpm [27]. These HFrEF patient profiles need to be treated with target β-blocker doses before initiating Ivabradine.

**Key Consensus Recommendations 2**

**Benefits of HR reduction in patients with CCS and HFrEF and role of Ivabradine**

- High RHR is often treated with β-blockers, Ivabradine or a combination of both, depending upon the tolerance of the patients or the presence of any contraindications to β-blockers in patients with CCS and HFrEF.
- Ivabradine has remarkable HR-lowering properties and is not associated with negative inotropic effects or hypotension.
- Ivabradine is a useful add on to first-line antianginal agents in patients with CCS and in patients with HFrEF who are on guideline- recommended/maximally tolerated doses of β-blockers and having sinus rates >70/min.
- Ivabradine can also be used for reducing HR in CCS and HFrEF patients who have contraindications for using β-blockers like asthma and chronic obstructive pulmonary disease.
- Ivabradine is indicated to reduce the heart rate in patients with HFrEF having low BP with high HR where β-blocker up-titration is not possible.
- In elderly patients, a lower dose of Ivabradine 2.5 mg can be considered to reduce the risk of bradycardia.

## 4 ACHIEVING TARGET HR: UNMET NEED IN CCS AND HFrEF

Similar to the targets given for BP management various guidelines have also provided targets for HR to be achieved in the management of CCS and HFrEF. The ACC 2021 expert consensus decision pathway suggested titration of Ivabradine to an HR of 50-60 bpm in patients with HFrEF [33]. In the settings of CCS, the ESC 2019 guidelines suggest an HR goal of 55-60 bpm [3]. Achieving these target HR goals is important in the management of patients with CCS and HFrEF. However, evidence from multiple studies suggests that the target HR is not achieved in the majority of patients with CCS or HFrEF even after receiving guideline-recommended therapy at optimal doses. The contributing factors for this unmet need of achieving target HR with available therapies may include inadequate attention to HR as a modifiable treatment target, physician’s inertia to up-titrate HR modulating treatments or the addition of other promising anti-anginal agents due to the fear of side effects [15]. Observations from Euro Heart Survey showed that more than half of the patient population had baseline HR >70 bpm and of these, around 40% of patients receiving β-blockers had RHR >70 bpm [15]. Similar trends were observed among patients included in the CLARIFY (prospeCtive observational LongitudinAl RegIstry oF patients with stable coronary artery disease) registry wherein 41% of patients receiving β-blockers had an HR >70 bpm [34, 35]. The scenario of HR management among CCS patients in India is even more worrying, with the average HR at baseline being significantly greater (76.1 *vs*. 68.0 bpm), the prevalence of HR ≥70 bpm higher (82.2% *vs*. 48.5%) and a very low proportion of patients achieving HR goal ≤60 bpm (2.5% *vs*. 22.9%) as compared to western cohorts [35]. Similar trends were observed in the Asian HF registry wherein Asians, including Indian patients, demonstrated a high average RHR (Asian population - 80 bpm and Indian population - 81 bpm) [36]. A study by Rao *et al.* has also reported a mean HR of 96 bpm at baseline among patients with systolic HF [37]. For achieving the desired HR goals, it is important to optimize the doses of HR-lowering agents, especially β-blockers, at the maximum tolerated doses. However, landmark trials and subsequent observational studies assessing β-blockers in patients with HFrEF showed low rates of patients achieving maximally tolerated target dose of β-blockers. Moreover, the study suggested a need for optimal dose titration of β-blockers to maintain optimal HR [38]. In a real-world study, sub optimal β-blocker dosage was attributed to the prevalent predictors of lower usage commonly witnessed in non-trial settings like low BP, presence of chronic obstructive pulmonary disease and older age. In the same study, there was also a significant proportion of patients having elevated HR despite being on guideline-directed β-blocker dose [39]. Also, in the post-discharge settings, the OPTIMIZE-HF (Organized Program to Initiate Lifesaving Treatment in Hospitalized Patients with Heart Failure) registry has reported a high mean HR (76 bpm) at the time of discharge among patients hospitalized with HF, in spite of a major proportion of the population being on β-blocker therapy. In addition, patients receiving β-blocker doses in the range of 50-99% of the target doses had high HR at discharge [40]. Similarly, the Norwegian Heart Failure Registry reported that a high proportion of patients who had an HR ≥70 bpm were not treated with or/did not tolerate the target dose of β-blockers. The study additionally suggested that increased efforts should be made to further increase the β-blocker dose and treatment with Ivabradine could be considered among patients with an HR ≥70 bpm [41]. It is also important to understand the relationship between achieving β-blocker dose *vs*. achieving HR targets. The OBTAIN (Outcomes of Beta-Blocker Therapy After MI) registry reported the impact of different doses of β-blocker on one-year post-MI survival in real-world settings wherein patients receiving >12.5% to 25% of the target dose had improved survival outcomes compared with those receiving no β-blockers and other β-blocker dose targets (>0% to 12.5%, >25%-50%, >50%) [42]. In another study where the relationship between HF mortality and β-blocker dose and RHR reported that RHR at visit 2 has independently improved prognosis but β-blocker dose did not. Moreover, the study demonstrated that in patients with an HR of 58-64 bpm, the prognosis was better as compared to those with an HR of >65 bpm [43]. Thus, although achieving the target β-blocker dose is important focusing also on achieving the target HR would be a promising approach to improve outcomes in patients with CCS and HFrEF.

**Key Consensus Recommendations 3**

**HR targets to be achieved and management options in CCS and HFrEF**

- In CCS patients, one should try to achieve an HR target of 55-60 bpm with the help of HR-lowering agents like β-blockers, calcium channel blockers and Ivabradine.
- In HFrEF patients, to maintain the cardiac output in the desired range, a higher HR target of 55-65 bpm should be considered with the help of HR-lowering agents like β-blockers and Ivabradine.
- Ideally, in HFrEF patients, the first choice of treatment for HR lowering should be β-blockers up-titrated to guidelinerecommended/ maximally tolerated doses.
- If β-blockers are not tolerated or there are contraindications for their use, in such cases of HFrEF Ivabradine should be preferred.

## 5 IVABRADINE/Β-BLOCKER COMBINATION FOR HR MANAGEMENT

**Fig. (1):**
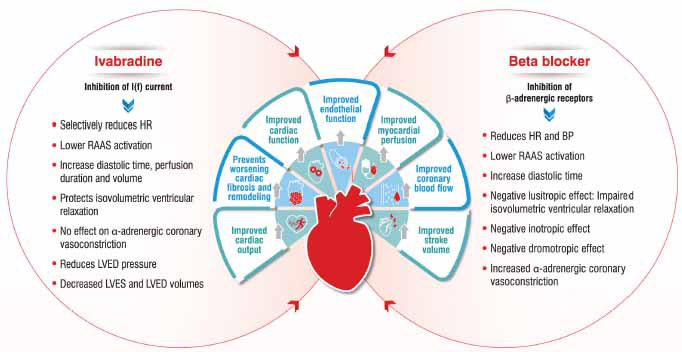
Synergic and complementary actions of Ivabradine and β-blocker Adapted from: Katsi *et al* [46] and Volterrani *et al* [47]. **Abbreviations:** HR: heart rate, BP: blood pressure, RAAS: renin-angiotensin-aldosterone system, LVED: left ventricular end-diastolic, LVES: left ventricular end-systolic.

Combination therapy has the advantage of better adherence to treatment, and reduced cost. Several previous studies have confirmed that fixed-dose combinations (FDCs) improved BP and cholesterol with similar rates of adverse events compared to individual components of drugs [44]. Additionally, FDCs in hypertension have been shown to reduce the risk of hospitalization with better treatment efficacy and greater BP reduction with a lower risk of CVD [45]. The Trivandrum Heart Failure Registry has also suggested that the use of combination therapy may help in better GDMT initiation and long-term adherence among patients with HFrEF [5]. In such scenario, FDC of Ivabradine and β-blockers may be particularly beneficial. Ivabradine acts on the hyperpolarization-activated cyclic nucleotide-gated channels and β-blockers reduce the sympathetic tones; therefore a combination of both these drugs can help to achieve better HR reduction than individual agents. A combination therapy of Ivabradine and β-blocker in small doses can be quite useful. The rationale for combining β-blockers and Ivabradine is that their cardiac actions are synergic and not limited to sinus node. Combining a β-blocker with Ivabradine can improve myocardial perfusion both at rest and during exercise which is associated with increased stroke volume and maintenance of cardiac output. Thus, Ivabradine perfectly complements the action of β-blockers (Fig. **1**) [46, 47]. Ivabradine is available as a FDC with metoprolol and carvedilol in India. Ivabradine/metoprolol FDC is indicated as substitution therapy for the symptomatic treatment of chronic stable angina with normal sinus rhythm who were already controlled on metoprolol and Ivabradine. Ivabradine/carvedilol FDC is indicated as substituted therapy in adult patients with normal sinus rhythm already controlled by Ivabradine and carvedilol taken concomitantly at the same doses level for the symptomatic treatment of chronic stable angina pectoris in CAD patients and for the treatment of chronic HF (NYHA class II-IV) with systolic dysfunction.

### 5.1 Clinical Evidence of Ivabradine/β-Blocker Combination in CCS

Evidence from various clinical trials supports the benefits of using a combination therapy of Ivabradine and β-blockers in the management of HR among patients with CCS [23]. Glezer *et al.* reported an effective reduction of HR and improvement in clinical outcomes, including a higher proportion of patients being angina-free with Ivabradine/β-blocker combination therapy as compared to β-blocker up-titration among patients with CAD [48]. Additionally, this combination showed good tolerability, safety and more pronounced clinical improvement in comparison to β-blocker up-titration [48]. Observations from another trial reported an effective reduction of HR, anginal attacks and nitrate use along with the enhanced quality of life (QOL) in patients with stable angina receiving Ivabradine/β-blocker combination therapy [49]. Furthermore, FDC of Ivabradine/metoprolol administered twice-daily over a period of 4-months was found beneficial in the reduction of HR, angina symptoms and improved Canadian Cardiovascular Society (CCS) score, mainly attributable to the increased medication adherence among patients with stable angina [50]. The benefits seen with this combination were regardless of age, CAD duration, CCS class, comorbidities, previous MI or history of revascularization [51]. Another study by Tardiff *et al.* showed that a combination of Ivabradine and β-blocker in patients with ischemic heart disease is more effective in improving exercise tolerance [52]. Similarly, a combination of Ivabradine and bisoprolol reduced angina symptom scores and improved ejection fraction in patients with stable angina when compared with bisoprolol alone [53]. Collectively, these evidences suggest that Ivabradine in combination with β-blockers is efficacious and well tolerated in CCS with the FDC providing an additional benefit of improving adherence.

### 5.2 Clinical Evidence of Ivabradine/β-Blocker Combination in HFrEF

As seen in CCS, clinical evidence from several studies has demonstrated the efficacy and safety of Ivabradine/β-blocker combination among patients with HFrEF. In a post-hoc analysis of the SHIFT study, the subgroup of patients receiving carvedilol with Ivabradine demonstrated improvements in CV outcomes and was found safe in HFrEF patients with an RHR ≥70 bpm [54]. In a study evaluating the effect of early treatment with Ivabradine combined with β-blockers *vs*. β-blockers alone in patients hospitalized with HFrEF (ETHIC-AHF), Ivabradine/β-blocker combination therapy demonstrated a significant reduction in HR, more proportion of patients achieving HR <70 bpm with improvement in LVEF at 12 months compared to those receiving β-blockers alone [55]. Furthermore, in the OPTIMIZE-HF program, combination therapy of β-blocker and Ivabradine started in hospitalized patients with HF resulted in 55% reduced risk of overall mortality and re-hospitalization and helped to achieve the target β-blocker dose [56]. In another study, chronic HFrEF patients treated with a combination of Ivabradine/carvedilol resulted in lower RHR, better exercise capacity and achieved higher doses of β-blocker more rapidly than patients without Ivabradine [57]. The CARVIVA-HF (CARVedilol, Ivabradine or their Combination on Exercise Capacity in Patients with Heart Failure) trial has demonstrated that the combination of carvedilol and Ivabradine improved exercise tolerance and QOL after 12 weeks of treatment [58]. Those patients who either fail to achieve a significant reduction in HR with β-blockers, decreased exercise capacity or with intolerance to higher doses of β-blockers will be ideal candidates for the addition of Ivabradine [59]. The FDC of Ivabradine/β-blocker can further help in reducing pill burden and improve adherence among HFrEF patients who are already fraught with polypharmacy [60].

**Key Consensus Recommendations 4**

**Role of Ivabradine/β-blocker combination in CCS and HFrEF**

- Ivabradine in combination with β-blockers effectively lowers HR and can exert other beneficial effects on the myocardium which are synergic and complementary to β-blockers.
- FDC therapy can help to reduce pill burden, improve compliance with less chances of prescription errors and adverse effects.
- A FDC of Ivabradine and β-blocker can be initiated in adult patients with normal sinus rhythm as substitution therapy who are already receiving and their HR is controlled with both the agents given individually.
- It is not recommended to use Ivabradine plus β-blockers FDC as a first-line treatment. A clinician should titrate the dose of β-blockers and Ivabradine individually and once the patient is stable then should opt for combination therapy.
- In patients receiving stable doses of Ivabradine and β-blockers other than metoprolol and carvedilol, prolonged release Ivabradine can be used in place of Ivabradine immediate-release formulation.
- In stabilized HFrEF patients with high HR at the time of discharge, Ivabradine and β-blocker can be co-administered and further uptitration can be done with careful monitoring of HR.

## 6 PROPOSED CONSENSUS ALGORITHMS

**Fig. (2):**
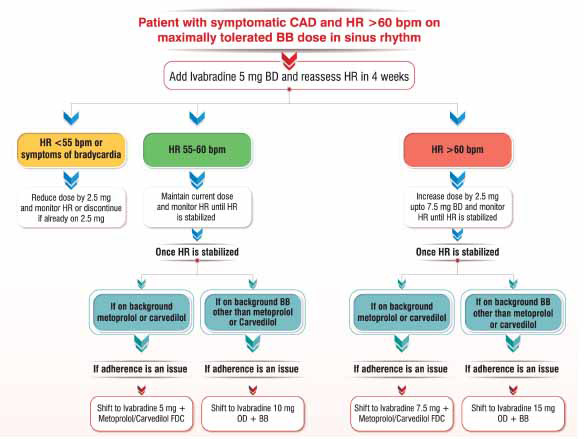
Proposed consensus algorithm for utilizing Ivabradine-based therapy in CCS/CAD **Abbreviations:** HR: heart rate, CAD: coronary artery disease, BB: β-blocker, BD: twice-daily, OD: once-daily, FDC: fixed-dose combination.

**Fig. (3):**
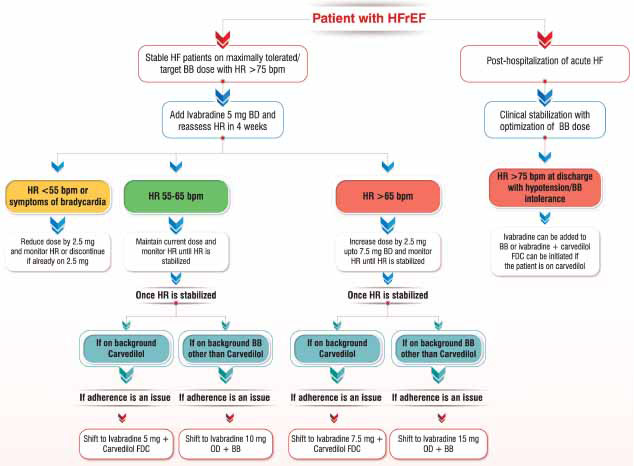
Proposed consensus algorithm for utilizing Ivabradine-based therapy in HFrEF **Abbreviations:** HR: heart rate, HFrEF: heart failure with reduced ejection fraction, BB: β-blocker, BD: twice-daily, OD: once-daily, FDC: fixed-dose combination

The comprehensive discussions between panel experts in all the meetings highlight the promising role of Ivabradine and its combination with β-blockers among patients with CCS and HFrEF. Based on the available evidence and guideline recommendations, this expert group has devised two algorithms that will help the treating physician to choose an appropriate Ivabradine-based therapy for managing patients with CCS (Fig. **2**) and HFrEF (Fig. **3**) in their day-to-day clinical practice.

## CONCLUSION

Heart rate is an important risk marker as well as a risk factor for CVD and its modulation can help to improve CV outcomes. A significant proportion of Indian patients with CCS and HFrEF have high RHR despite using β-blocker. Moreover, the up-titration of β-blockers to higher doses in patients with CCS and HFrEF may not always be achieved, mainly due to intolerance. Ivabradine is a well-established add on therapy available as twice-daily and once-daily prolonged release formulation which significantly reduces HR and exerts other beneficial effects on the myocardium. The combination of Ivabradine/β-blocker provides synergistic and complimentary effects in CCS and HFrEF thereby significantly reducing HR, and improve QOL with FDC having the potential to reduce pill burden and improve adherence.

## LIST OF ABBREVIATIONS

HR: Heart Rate
HFrEF: Heart Failure with Reduced Ejection Fraction
BB: β-Blocker
BD: Twice-Daily
OD: Once-Daily
FDC: Fixed-Dose Combination

## References

[1] Roth G.A., Mensah G.A., Johnson C.O. (2020). Global burden of cardiovascular diseases and risk factors, 1990–2019.. J. Am. Coll. Cardiol..

[2] Sreeniwas K.A., Sinha N. (2020). Cardiovascular disease in India: A 360 degree overview.. Med. J. Armed Forces India.

[3] Knuuti J., Wijns W., Saraste A. (2020). 2019 ESC Guidelines for the diagnosis and management of chronic coronary syndromes.. Eur. Heart J..

[4] Groenewegen A., Rutten F.H., Mosterd A., Hoes A.W. (2020). Epidemiology of heart failure.. Eur. J. Heart Fail..

[5] Harikrishnan S., Jeemon P., Ganapathi S. (2021). Five-year mortality and readmission rates in patients with heart failure in India: Results from the Trivandrum heart failure registry.. Int. J. Cardiol..

[6] Jensen M.T. (2019). Resting heart rate and relation to disease and longevity: past, present and future.. Scand. J. Clin. Lab. Invest..

[7] Benetos A., Rudnichi A., Thomas F., Safar M., Guize L. (1999). Influence of heart rate on mortality in a French population: role of age, gender, and blood pressure.. Hypertension.

[8] Jouven X., Zureik M., Desnos M., Guérot C., Ducimetière P. (2001). Resting heart rate as a predictive risk factor for sudden death in middle-aged men.. Cardiovasc. Res..

[9] Hamill V., Ford I., Fox K. (2015). Repeated heart rate measurement and cardiovascular outcomes in left ventricular systolic dysfunction.. Am. J. Med..

[10] Kaul U., Wander G.S., Sinha N. (2020). Self-blood pressure measurement as compared to office blood pressure measurement in a large Indian population; the India Heart Study.. J. Hypertens..

[11] Rao D., Balagopalan J.P., Sharma A., Chauhan V.C., Jhala D. (2015). BEAT survey: A cross-sectional study of resting heart rate in young (18-55 Year) hypertensive patients.. J. Assoc. Physicians India.

[12] Sengupta S.P., Burkule N., Bansal M. (2021). Normative values of cardiac chamber dimensions and global longitudinal strain in Indians: The Indian normative data of echocardiography analyzed (INDEA) study.. Int. J. Cardiovasc. Imaging.

[13] Padmanabhan T.N.C., Dani S., Chopra V.K., Guha S., Vasnawala H., Ammar R. (2014). Prevalence of sympathetic overactivity in hypertensive patients – A pan India, non-interventional, cross sectional study.. Indian Heart J..

[14] Aune D, Sen A, ó’Hartaigh B (2017). Resting heart rate and the risk of cardiovascular disease, total cancer, and all-cause mortality – A systematic review and dose–response meta-analysis of prospective studies.. Nutr. Metab. Cardiovasc. Dis..

[15] Daly C.A., Clemens F., Lopez Sendon J.L. (2010). Inadequate control of heart rate in patients with stable angina: Results from the European Heart Survey.. Postgrad. Med. J..

[16] Fox K., Ford I., Steg P.G., Tendera M., Robertson M., Ferrari R. (2008). Heart rate as a prognostic risk factor in patients with coronary artery disease and left-ventricular systolic dysfunction (BEAUTIFUL): A subgroup analysis of a randomised controlled trial.. Lancet.

[17] Kurgansky K.E., Schubert P., Parker R. (2020). Association of pulse rate with outcomes in heart failure with reduced ejection fraction: A retrospective cohort study.. BMC Cardiovasc. Disord..

[18] Habal M.V., Liu P.P., Austin P.C. (2014). Association of heart rate at hospital discharge with mortality and hospitalizations in patients with heart failure.. Circ. Heart Fail..

[19] Ferrari R., Fox K. (2016). Heart rate reduction in coronary artery disease and heart failure.. Nat. Rev. Cardiol..

[20] Koruth J.S., Lala A., Pinney S., Reddy V.Y., Dukkipati S.R. (2017). The clinical use of ivabradine.. J. Am. Coll. Cardiol..

[21] Tse S., Mazzola N. (2015). Ivabradine (Corlanor) for heart failure: The first selective and specific I f Inhibitor.. P&T.

[22] Mullasari A., Mahajan A., Chanana B.B. (2020). Efficacy and safety of ivabradine once-daily prolonged-release versus twice-daily immediate-release formulation in patients with stable chronic heart failure with systolic dysfunction: A randomized, double-blind, phase 3 non-inferiority (PROFICIENT) study.. Cardiol. Ther..

[23] Kaski J.C., Gloekler S., Ferrari R. (2018). Role of ivabradine in management of stable angina in patients with different clinical profiles.. Open Heart.

[24] Fox K., Ford I., Steg P.G., Tendera M., Robertson M., Ferrari R. (2009). Relationship between ivabradine treatment and cardiovascular outcomes in patients with stable coronary artery disease and left ventricular systolic dysfunction with limiting angina: A subgroup analysis of the randomized, controlled BEAUTIFUL trial.. Eur. Heart J..

[25] Fox K., Ford I., Steg P.G., Tardif J.C., Tendera M., Ferrari R. (2014). Ivabradine in stable coronary artery disease without clinical heart failure.. N. Engl. J. Med..

[26] Giavarini A., de Silva R. (2016). The role of ivabradine in the management of angina pectoris.. Cardiovasc. Drugs Ther..

[27] McDonagh T.A., Metra M., Adamo M. (2021). 2021 ESC guidelines for the diagnosis and treatment of acute and chronic heart failure.. Eur. Heart J..

[28] Swedberg K., Komajda M., Böhm M. (2010). Ivabradine and outcomes in chronic heart failure (SHIFT): A randomised placebo-controlled study.. Lancet.

[29] Swedberg K., Komajda M., Böhm M. (2012). Effects on outcomes of heart rate reduction by ivabradine in patients with congestive heart failure: is there an influence of beta-blocker dose?: Findings from the SHIFT (Systolic Heart failure treatment with the I(f) inhibitor ivabradine Trial) study.. J. Am. Coll. Cardiol..

[30] Borer J.S., Böhm M., Ford I. (2012). Effect of ivabradine on recurrent hospitalization for worsening heart failure in patients with chronic systolic heart failure: the SHIFT Study.. Eur. Heart J..

[31] Komajda M., Tavazzi L., Swedberg K. (2016). Chronic exposure to ivabradine reduces readmissions in the vulnerable phase after hospitalization for worsening systolic heart failure: A post-hoc analysis of SHIFT.. Eur. J. Heart Fail..

[32] Heidenreich P.A., Bozkurt B., Aguilar D. (2022). 2022 AHA/ACC/HFSA guideline for the management of heart failure: A report of the american college of cardiology/american heart association joint committee on clinical practice guidelines.. Circulation.

[33] Maddox T.M., Januzzi J.L., Allen L.A. (2021). 2021 update to the 2017 ACC expert consensus decision pathway for optimization of heart failure treatment: answers to 10 pivotal issues about heart failure with reduced ejection fraction.. J. Am. Coll. Cardiol..

[34] Steg P.G., Ferrari R., Ford I. (2012). Heart rate and use of beta-blockers in stable outpatients with coronary artery disease.. PLoS One.

[35] Kau L.U., Natrajan S., Dalal J., Saran R.K. (2017). Prevalence and control of cardiovascular risk factors in stable coronary artery outpatients in India compared with the rest of the world: An analysis from international CLARIFY registry.. Indian Heart J..

[36] Lam C.S.P., Teng T.H.K., Tay W.T. (2016). Regional and ethnic differences among patients with heart failure in Asia: the Asian sudden cardiac death in heart failure registry.. Eur. Heart J..

[37] Rao M.S., Mandal S. (2017). Epidemiologic surveillance on quality of life in patients with systolic heart failure after treatment with the selective heart rate inhibitor ivabradine.. J. Pract. Cardiovasc. Sci..

[38] Bhatt A.S., DeVore A.D., DeWald T.A., Swedberg K., Mentz R.J. (2017). Achieving a maximally tolerated β-blocker dose in heart failure patients.. J. Am. Coll. Cardiol..

[39] Ibrahim N.E., Gaggin H.K., Turchin A. (2019). Heart rate, beta-blocker use, and outcomes of heart failure with reduced ejection fraction.. Eur. Heart J. Cardiovasc. Pharmacother..

[40] DeVore A.D., Mi X., Mentz R.J. (2016). Discharge heart rate and β-blocker dose in patients hospitalized with heart failure: Findings from the OPTIMIZE-HF registry.. Am. Heart J..

[41] Eriksen-Volnes T, Westheim A, Gullestad L, Slind EK, Grundtvig M (2020). β-blocker doses and heart rate in patients with heart failure: Results from the national norwegian heart failure registry. Biomed. Hub.

[42] Goldberger JJ, Subačius H, Marroquin OC (2021). One-year landmark analysis of the effect of beta-blocker dose on survival after acute myocardial infarction.. J. Am. Heart Assoc..

[43] Cullington D., Goode K.M., Clark A.L., Cleland J.G.F. (2012). Heart rate achieved or beta-blocker dose in patients with chronic heart failure: Which is the better target?. Eur. J. Heart Fail..

[44] Webster R., Murphy A., Bygrave H., Ansbro É., Grobbee D.E., Perel P. (2020). Implementing fixed dose combination medications for the prevention and control of cardiovascular diseases.. Glob. Heart.

[45] Rea F., Corrao G., Merlino L., Mancia G. (2018). Early cardiovascular protection by initial two-drug fixed-dose combination treatment *vs*. monotherapy in hypertension.. Eur. Heart J..

[46] Katsi V., Skalis G., Kallistratos M.S. (2019). Ivabradine and metoprolol in fixed dose combination: When, why and how to use it.. Pharmacol. Res..

[47] Volterrani M., Iellamo F. (2016). Complementary and synergic role of combined beta-blockers and ivabradine in patients with chronic heart failure and depressed systolic function: A new therapeutic option?. Card. Fail. Rev..

[48] Glezer M., Vasyuk Y., Karpov Y. (2018). Efficacy of ivabradine in combination with beta-blockers versus uptitration of beta-blockers in patients with stable angina (CONTROL-2 Study).. Adv. Ther..

[49] Werdan K., Ebelt H., Nuding S., Höpfner F., Hack G., Müller-Werdan U. (2012). Ivabradine in combination with beta-blocker improves symptoms and quality of life in patients with stable angina pectoris: Results from the ADDITIONS study.. Clin. Res. Cardiol..

[50] Divchev D., Stöckl G. (2017). Treatment of stable angina with a new fixed-dose combination of ivabradine and metoprolol: Effectiveness and tolerability in routine clinical practice.. Cardiol. Ther..

[51] Divchev D., Stöckl G. (2019). Effectiveness and impact on adherence of a new fixed-dose combination of ivabradine and metoprolol in a wide range of stable angina patients in real-life practice.. Cardiol. Ther..

[52] Tardif J.C., Ponikowski P., Kahan T. (2013). Effects of ivabradine in patients with stable angina receiving β-blockers according to baseline heart rate: an analysis of the ASSOCIATE study.. Int. J. Cardiol..

[53] Amosova E., Andrejev E., Zaderey I., Rudenko U., Ceconi C., Ferrari R. (2011). Efficacy of ivabradine in combination with Beta-blocker versus uptitration of Beta-blocker in patients with stable angina.. Cardiovasc. Drugs Ther..

[54] Bocchi E.A., Böhm M., Borer J.S. (2015). Effect of combining ivabradine and β-blockers: Focus on the use of carvedilol in the shift population.. Cardiology.

[55] Hidalgo FJ, Carrasco F, Castillo JC (2017). Early therapy with beta blockers plus ivabradine versus beta blockers alone in patients hospitalised with heart failure and reduced ejection fraction (ETHICAHF Study): Results at one-year follow-up.. Int J Clin Cardiol.

[56] Lopatin Y.M., Cowie M.R., Grebennikova A.A. (2018). Optimization of heart rate lowering therapy in hospitalized patients with heart failure: insights from the optimize heart failure care program.. Int. J. Cardiol..

[57] Bagriy A.E., Schukina E.V., Samoilova O.V. (2015). Addition of ivabradine to β-blocker improves exercise capacity in systolic heart failure patients in a prospective, open-label study.. Adv. Ther..

[58] Volterrani M., Cice G., Caminiti G. (2011). Effect of carvedilol, ivabradine or their combination on exercise capacity in patients with heart failure (the CARVIVA HF trial).. Int. J. Cardiol..

[59] Zugck C., Martinka P., Stöckl G. (2014). Ivabradine treatment in a chronic heart failure patient cohort: Smptom reduction and improvement in quality of life in clinical practice.. Adv. Ther..

[60] Bhatt A.S., Choudhry N.K. (2021). Evidence-based prescribing and polypharmacy for patients with heart failure.. Ann. Intern. Med..

